# Study on risk factors, bacterial species, and drug resistance of acute pyelonephritis associated with ureteral stent after percutaneous nephrolithotomy

**DOI:** 10.1007/s10096-020-04050-z

**Published:** 2020-10-09

**Authors:** Guo Jiang, Jiang Li, He Long, Chen Qiulin, Ren Jin, Yang Yaodong, Dong Xingyou, Zhao Jiang, Zhang Zhenyang

**Affiliations:** 1 Department of Urology, Anyue People’s Hospital of Ziyang City, Sichuan Province, Ziyang, 642300 China; 2 Department of Urology, Chongqing Hechuan Hongren Hospital, Chongqing, 401520 China

**Keywords:** Percutaneous nephrolithotomy, Ureteral stent, Acute pyelonephritis, Infection, Bacterial strain, Drug sensitivity, Drug resistance

## Abstract

The purpose of this study is to explore the risk factors, bacterial species, and drug resistance of acute pyelonephritis (AP) associated with ureteral stent after percutaneous nephrolithotomy (PCNL) and to provide reference for clinical intervention. The clinical data of 415 patients with indwelling ureteral stent after PCNL from December 2016 to May 2019 were analyzed retrospectively. The patients were divided into infection group (*n* = 54) and non-infection group (*n* = 361) according to whether patients had AP. Patients’ clinical data, blood and urine bacterial culture, and drug sensitivity were collected and analyzed. The incidence of AP associated with ureteral stent after PCNL was 13.01% and diabetes mellitus (*P* = 0.001), postoperative stone residue (*P* = 0.002), urinary leucocytes ≥ 100/HP (*P* = 0.018), positive urine culture results (*P* = 0.001), ureteral stent retention time ≥ 8 weeks (*P* = 0.004), and high S.T.O.N.E. score (*P* = 0.014) are independent risk factors for it. *Escherichia coli* (40.54%, 47.82%), *Klebsiella pneumoniae* (16.21%, 15.21%), *Pseudomonas aeruginosa* (10.81%, 4.34%), *Enterococcus faecalis* (21.6%, 19.56%), and epidermis Staphylococci (10.81%, 13.33%) are the main pathogens in blood and urine. The main sensitive drugs of pathogenic bacteria are imipenem, meropenem, tigecycline, piperacillin/tazobactam, ceftazidime, linezolid, teicoplanin, levofloxacin, vancomycin, tigecycline, etc., while levofloxacin, norfloxacin, penicillin G, first, and second-generation cephalosporins showed a strong drug resistance rate (> 70%). This study found that diabetes, postoperative stone residuals, urinary leukocytes ≧ 100 cells/HP, positive urine culture results, ureteral stent indwelling time ≧ 8 weeks, and high S.T.O.N.E. score were independent of AP associated with ureteral stent after PCNL risk factors and *Escherichia coli* is the main pathogenic bacteria and shows drug resistance.

## Introduction

Kidney stones are a common and frequently occurring disease of the urinary system. The incidence is 10.34% in men and 6.62% in women [1]. Percutaneous nephrolithotomy (PCNL) is the main treatment method for kidney stones [2, 3]. PCNL has been used as an important minimally invasive method for the treatment of kidney stones because of its small trauma and rapid recovery [2, 3]. Indwelling double J-tube after PCNL can provide advantages such as urinary tract obstruction, drainage of urine, and protection of renal function [4, 5]. But after PCNL operation, the ureteral stent can cause the patient’s waist and abdomen discomfort, bladder irritation, hematuria, stent tube displacement, and other adverse events, especially urinary tract infection is one of the more common complications [4, 5]. There are some data suggesting an association between indwelling ureteral stents and urinary tract infections, including a recent prospectively performed study reporting an 11% incidence of UTIs in stented patients [4, 5]. In clinic, antibiotics are mainly used to treat acute pyelonephritis (AP) associated with ureteral stent after PCNL. However, clinical observations have shown that this infection is not effectively treated with ordinary second-generation cephalosporins and quinolone antibacterials such as levofloxacin, which indicates that the pathogenic bacteria of this kind of infection are drug resistance and difficult to treat. Accordingly, this study retrospectively analyzed the clinical data of 415 patients with indwelling ureteral stent after PCNL treatment in our hospital from December 2016 to May 2019 to explore the risk factors, bacterial strains, and drug susceptibility of AP associated with ureteral stent after PCNL, and provide a reference for clinical intervention.

## Materials and methods

### Case collection and establishment of diagnostic criteria for AP

From December 2016 to May 2019, 415 cases of PCNL patients with ureteral stent (retention time ≥ 4 weeks) were collected. According to whether the patients had AP, they were divided into infection group (*n* = 54) and non-infection group (*n* = 361). Inclusion criteria: (1) first diagnosis of kidney stones by abdominal ultrasound, CT, or intravenous pyelography; (2) first kidney stones undergoing PCNL and indwelling ureteral stent; (3) patients and family members agreed with the study and signed informed consent. Exclusion criteria: (1) patients with contraindications for renal calculi operation; (2) patients with severe cardiopulmonary dysfunction; (3) patients with immune system disease; (4) patients with blood system disease; (5) patients with severe liver and kidney dysfunction; (6) patients with previous history of renal calculi operation; (7) patients with pyonephrosis; (8) patients with other organ infection and febrile diseases. Diagnostic criteria of AP: (1) the patient has acute pain in one or both sides of the back (the pain can radiate to the waist, abdomen, and/or groin area); (2) percussion pain or tenderness of costal ridge angle; (3) body temperature ≥ 38.0 °C; (4) blood routine examination showed that leukocyte was more than 10.0 × 10^9^/L; (5) urine routine white blood cell count > 5/HP and/or middle urine culture positive, and colony count ≥ 105 CFU/mL. It can be diagnosed as AP if it is greater than or equal to the three of the above 5 [6–8]. This study was approved by the Ethics Committee of Anyue People’s Hospital of Ziyang City. All the patients and their families in the study signed the relevant informed consent.

### PCNL operation mode and clinical data collection

The operation procedure and method of PCNL were performed in accordance with the previous literature standards [9–11]. The data of gender, age, BMI, history of basic diseases, location of affected kidney, S.T.O.N.E. score, operation time, stone residue after operation, urine leukocyte count, ureteral stent retention time, operation channel size, blood, and urine bacterial culture were collected for statistical analysis. The S.T.O.N.E. score of renal calculi refers to the previous report [12, 13].

### Bacterial culture, identification, and drug sensitivity test

Patients’ blood and mid-section urine were collected for bacterial culture and identification. MicroScan WalkAway 40 bacterial identification instrument from German Siemens Company was used for bacterial strain identification and bacterial culture experiment, and bacterial drug sensitivity test. Bacterial culture, identification, and drug susceptibility experiments were completed with the assistance of the clinical laboratory of our hospital.

### Statistical analysis

SPSS 19.0 statistical analysis software is used for data analysis. The data of measurement data are expressed in mean ± standard deviation, analyzed by *t* test; count data is expressed as a percentage (%) and analyzed by *χ*^2^ test. The factors with statistical significance were analyzed by logistic regression analysis, and the regression coefficient (*β*), relative risk ratio (or), and 95% confidence interval (95% CI) were calculated. The difference was statistically significant (*P* < 0.05).

## Results

### Single factor analysis of risk factors for AP associated with ureteral stent after PCNL

According to the exclusion and inclusion criteria, a total of 415 patients were included in the study, 54 cases in the infected group and 361 cases in the non-infected group. There were 192 women (46.26%) and 223 men (53.73%). The incidence of AP associated with ureteral stent after PCNL is 13.01%. As shown in Table 1, we found that the risk factors of AP associated with ureteral stent after PCNL were gender, diabetes mellitus, stone residue after operation, white blood cells in urine ≥ 100/HP, retention time of ureteral stent ≥ 8 weeks, positive urine culture result, and high S.T.O.N.E. score (especially the large stone size, severe obstruction, and multiple kidney calices involved). However, age, BMI, location of affected kidney, history of hypertension, history of coronary heart disease, history of chronic renal insufficiency, and size of operation channel were not risk factors of AP associated with ureteral stent after PCNL.

**Table 1:** single factor analysis of risk factors of AP associated with ureteral stent after PCNL

Parameter		Infection group (*n* = 54)	Non-infected group (*n* = 361)	*P* value
Age	≧ 65	30	190	
	< 65	24	171	0.688
BMI (kg/m^2^)		26.81 ± 4.32	25.25 ± 4.57	0.142
Gender	Male	20	203	
	Female	34	158	0.007
Location of affected kidney	Left	21	196	
	Right	33	165	0.296
Operation time (min)		67.01 ± 21.21	64.14 ± 24.57	0.124
Hypertension	Yes	19	85	
	No	35	276	0.095
Coronary heart disease	Yes	16	92	
	No	38	269	0.467
Diabetes	Yes	32	89	
	No	22	272	< 0.001
Chronic renal insufficiency	Yes	26	129	
	No	28	232	0.079
Residual calculus after operation	Yes	23	73	
	No	31	288	< 0.001
Urine leukocyte (a/HP)	≧ 100	33	117	
	< 100	21	244	< 0.001
Retention time of ureteral stent (week)	≧ 8 week	29	101	
	4–8 week	25	260	< 0.001
Operation channel size	< 22F	28	169	
	≧ 22F	25	192	0.413
Positive urine culture result	Positive	40	76	
	Negative	14	285	< 0.001
S.T.O.N.E. score		7.62 ± 1.45	7.12 ± 1.45	< 0.001
S		2.89 ± 0.75	2.51 ± 0.81	0.001
T		1.49 ± 0.62	1.42 ± 0.87	0.156
O		1.53 ± 0.41	1.39 ± 0.45	< 0.001
N		2.07 ± 0.61	1.85 ± 0.79	< 0.001
E		1.55 ± 0.41	1.53 ± 0.52	0.201

*S*, stone size; *T*, tract length; *O*, obstruction degree; *N*, number of involved calices; *E*, essence or stone density

### Multiple logistic regression analysis of risk factors of AP associated with ureteral stent after PCNL

As shown in Table 2, multiple logistic regression analysis showed that diabetes mellitus (*P* = 0.001), postoperative stone residue (*P* = 0.002), urinary leucocytes ≥ 100/HP (*P* = 0.018), positive urine culture results (*P* = 0.001), ureteral stent retention time ≥ 8 weeks (*P* = 0.004), and high S.T.O.N.E. score (*P* = 0.014, especially the large stone size, severe obstruction, and multiple kidney calices involved) are independent risk factors for AP associated with ureteral stent after PCNL. Among them, diabetes history, postoperative stone residue, positive urine culture results, and ureteral stent retention time ≥ 8 weeks had the highest impact.

**Table 2:** Multiple logistic regression analysis of risk factors of AP associated with ureteral stent after PCNL

Risk factors	*β*	*P* value	OR value	95% CI
Gender	0.376	0.413	1.241	0.914, 1.853
Diabetes	1.504	0.001*	3.318	2.718, 5.279
Residual calculus after operation	1.378	0.002*	3.087	2.902,5.021
Urine leukocytes (≧ 100/HP)	0.955	0.018*	2.171	1.685, 3.245
Ureteral stent (indwelling ≧ 8 weeks)	1.055	0.004*	2.017	1.611, 3.247
Positive urine culture	1.567	0.001*	3.628	2.845, 6.214
S.T.O.N.E. score	1.131	0.014*	1.441	1.024, 2.254
S	0.887	0.021*	1.032	0.845, 1.769
0	1.457	0.003*	1.845	1.147, 2.868
N	1.378	0.006*	1.672	1.274, 2.419

*S*, stone size; *T*, tract length; *O*, obstruction degree; *N*, number of involved calices; *E*, essence or stone density

### Bacterial culture and drug sensitivity analysis of urine and blood samples of patients with AP associated with ureteral stent after PCNL

As shown in Table 3, there were 54 blood and urine samples, 37 blood culture positive samples (68.51%), and 45 urine culture positive samples (85.18%). As shown in Table 3, in the culture components of blood and urine bacteria, Gram-negative bacteria are mainly *Escherichia coli*, *Klebsiella pneumoniae*, and *Pseudomonas aeruginosa*, and Gram-positive bacteria are mainly *Enterococcus faecalis* and *Staphylococcus epidermidis*. As shown in Table 4, we analyzed the drug resistance of bacteria in the blood and urine of patients and found that the main sensitive drugs for pathogenic bacteria (drug resistance rate < 15%) are imipenem, meropenem, tigecycline, piperacillin/tazobactam, ceftazidime, linezolid, teicoplanin, levofloxacin star, vancomycin, tigecycline, etc., but levofloxacin, norfloxacin, penicillin G, compound sinomine, and second-generation cephalosporins showed strong drug resistance (> 70%).

**Table 3:** Analysis of bacterial species in urine and blood samples of patients with AP associated with ureteral stent after PCNL (*n* = 54, %)

Specimen	*n* (54)	*Escherichia coli*	*Enterococcus faecalis*	*Staphylococcus epidermidis*	*Klebsiella pneumoniae*	*Pseudomonas aeruginosa*	Fungus
Blood	37 (68.51%)	15 (40.54%)	8 (21.62%)	4 (10.81%)	6 (16.21%)	4 (10.81%)	0 (0%)
Urine	46 (85.18%)	22 (47.82%)	9 (19.56%)	5 (13.33%)	7 (15.21%)	2 (4.34%)	1 (2.17%)

**Table 4:** Analysis of urine and blood bacterial drug resistance rates of AP associated with double J-tube after PCNL (*n* = 83, %)

	*Escherichia coli*	*Enterococcus faecalis*	*Staphylococcus epidermidis*	*Klebsiella pneumoniae*	*Pseudomonas aeruginosa*
Penicillin G	100%	100%	95.32%	100%	100%
Ampicillin	93.97%	100%	81.92%	100%	93.97%
Sulfamethoxazole compound	86.74%	92.77%	85.54%	96.38%	100%
Cefepime	81.92%	85.54%	89.15%	93.97%	95.18%
Ceftiofur	91.56%	74.69%	83.13%	85.54%	87.95%
Ceftriaxone	72.28%	77.10%	71.08%	79.51%	83.13%
Ciprofloxacin	42.16%	36.14%	39.75%	57.83%	50.60%
Norfloxacin	69.87%	71.08%	61.44%	53.01%	63.85%
Levofloxacin	78.31%	74.98%	66.26%	81.92%	85.54%
Amoxicillin-clavulanic acid	37.34%	32.53%	31.32%	46.51%	53.01%
Amikacin	9.63%	13.25%	8.43%	12.04%	10.46%
Piperacillin/tazobactam	6.02%	7.23%	9.63%	13.08%	14.45%
Cefoperazone/sulbactam sodium	15.66%	13.25%	18.07%	16.86%	12.04%
Meropenem	2.40%	1.20%	0%	0%	3.61%
Imipenem	0%	0%	0%	0%	0%
Ceftazidime	23.25%	19.27%	21.68%	18.07%	24.09%
Furantoin	36.14%	39.75%	34.93%	48.19%	56.63
Linezolid	0%	0%	0%	0%	0%
Teicoplanin	0%	0%	0%	0%	0%
Levofloxacin	0%	0%	0%	0%	0%
Vancomycin	0%	0%	0%	0%	2.40%
Tigecycline	0%	0%	0%	0%	0%

## Discussion

Ureteral stent is the most commonly used medical implant for urological surgery. It is widely used in clinical practice because it can relieve urinary tract obstruction, drain urine, and protect renal function [14, 15]. After PCNL operation, ureteral stent is usually retained for 4–6 weeks, but some patients need to keep ureteral stent for a long time because of the complex condition. During the indwelling of ureteral stent after PCNL, AP associated with ureteral stent after PCNL is a more common complication, and some severe cases can induce retrograde urogenic sepsis endangering the life of patients [16–18]. Previous studies have suggested that the incidence of bacteriuria and urinary tract infection related to ureteral stent is 11–45% [4, 5]. In this study, we found that the incidence of AP associated with ureteral stent after PCNL was approximately 13.01%. The results are similar to previous reports, suggesting that the incidence of AP associated with ureteral stent after PCNL is higher, and urologists need to pay more attention.

Studies suggest that infections are a more common complication after PCNL. Long operation time, intraoperative bleeding, excessive intrapelvic pressure, and urinary tract obstruction are all risk factors for postoperative infection of PCNL [19–21]. In this study, we found that diabetes mellitus, postoperative stone residue, urinary leucocytes ≥ 100/HP, positive urine culture results, ureteral stent retention time ≥ 8 weeks, and high S.T.O.N.E. score (especially the large stone size, severe obstruction, and multiple kidney calices involved) are independent risk factors for AP associated with ureteral stent after PCNL. The possible reasons for the findings are the following: (1) the immune defense of diabetes patients is reduced, and the blood glucose of some patients is not well controlled for a long time, and the whole body glucose metabolism is disordered, which is conducive to the invasion, production, and reproduction of bacteria. In addition, it is difficult to remove the residual bacteria in diabetic patients before PCNL operation. The ureteral stent is more conducive to the continuous colonization and reproduction of bacteria [22, 23]; (2) as a foreign body, the ureteral stent is accompanied by the friction damage of the ureteral stent to the ureteral mucosa when the human body moves, which destroys the defense mechanism of the urinary tract epithelial system. In addition, the long-term retention of the ureteral stent (≥ 8 weeks) can form stone crystals on the surface of the ureteral stent, which is conducive to the adsorption, growth, and reproduction of bacteria [4, 5]; (3) PCNL was positive for urine culture before surgery, and turned negative after active anti-infective treatment. However, for some complex stones with high S.T.O.N.E. scores (especially the large stone size, severe obstruction, and multiple kidney calices involved), residual stones are present after surgery, and bacterial colonies are often present in the residual stones. These bacterial colonies, under the natural protective barrier provided by the bacterial biofilm, prevent the killing of bacteria by antibacterial drugs and eventually lead to bacterial reproduction and infection recurrence [4, 5, 21].

It is suggested that *Escherichia coli*, *Enterococcus*, *Klebsiella pneumoniae*, *Proteus mirabilis*, and *Pseudomonas aeruginosa* are the common pathogens of urinary tract infection induced by urinary stones [24, 25]. In this study, we found that among the pathogens causing AP associated with ureteral stent after PCNL, Gram-negative bacteria were mainly *Escherichia coli*, *Klebsiella pneumoniae*, and *Pseudomonas aeruginosa*, and Gram-positive bacteria are mainly *Enterococcus faecalis* and *Staphylococcus epidermidis*. In recent years, the unreasonable use or abuse of antibiotics in China, the drug resistance of pathogenic bacteria is becoming more and more serious, especially in patients with kidney stones and urinary tract infection. In China, the resistance rate of *E. coli* to quinolones (levofloxacin) and gentamycin was close to 50%, and the resistance rate to cephalosporins increased gradually, among which 79.2% and 66.5% to the first and second-generation cephalosporins respectively [26–28]. In this study, we also found that the pathogens of AP associated with ureteral stent after PCNL had strong resistance to quinolones (levofloxacin) and the first and second-generation cephalosporins (the resistance rate was more than 50%). These results suggest that quinolones (levofloxacin) and the first and second-generation cephalosporins are no longer suitable for the treatment of AP associated with ureteral stent after PCNL. In this study, we analyzed the pathogenic bacteria of AP associated with ureteral stent after PCNL. We found that the pathogenic bacteria were sensitive to β-lactam combined enzyme inhibitors (piperacillin/tazobactam) and aminoglycoside antibiotics (amikacin), and the resistance rate was less than 15%. Therefore, for mild to moderate infections, β-lactam combined with enzyme inhibitors (piperacillin/tazobactam) and aminoglycoside antibiotics (amikacin) are highly sensitive to pathogens and can be used as the first choice of drugs, but aminoglycoside antibiotics (amikacin) should be used carefully due to renal toxicity and ototoxicity. In addition, we also found that the pathogenic bacteria were sensitive to carbapenems (meropenem, imipenem), glycyl tetracyclines (tigecycline), azolidone antibiotics (linezolid), glycopeptide antibiotics (teicoplanin, vancomycin), and other drugs, and the resistance rate was less than 10%. Therefore, for severe infections, we recommend the use of this class of restricted antibiotics for anti-infective treatment in the absence of susceptibility to the pathogen.

In conclusion, diabetes mellitus, postoperative stone residue, leukocyte count ≥ 100/HP, positive urine culture result, ureteral stent retention time ≥ 8 weeks, and high S.T.O.N.E. score (especially the large stone size, severe obstruction, and multiple kidney calices involved) were independent risk factors of AP associated with ureteral stent retention after PCNL. *Escherichia coli*, *Enterococcus faecalis*, *Staphylococcus epidermidis*, *Klebsiella pneumoniae*, and *Pseudomonas aeruginosa* are the main pathogens of AP associated with double J-tube after PCNL. The pathogenic bacteria were sensitive to amikacin, β-lactam combined with enzyme inhibitor compound, carbapenems, and other drugs.
